# Exploring Neural Idiosyncrasies in Response to Autonomous Sensory Meridian Response Videos: Naturalistic Functional Magnetic Resonance Imaging Study of Stress and Sensory Processing

**DOI:** 10.2196/68586

**Published:** 2025-07-29

**Authors:** Hye Eun Lee, Sungbin Youk, Yoon Esther Lee, Musa Malik, René Weber

**Affiliations:** 1 Division of Communication & Media, College of Social Science, Ewha Womans University, Seoul, Republic of Korea; 2 Newhouse School of Public Communications, Syracuse University, Syracuse, NY, United States; 3 Department of Communication, Media Neuroscience Lab, University of California, 4005 Social Sciences & Media Studies, Santa Barbara, CA, United States, 1 8058932156; 4 Department of Psychological and Brain Sciences, University of California, Santa Barbara, CA, United States

**Keywords:** ASMR videos, autonomous sensory meridian response, stress-relief treatment, mental health, media therapy, fMRI, magnetic resonance imaging, naturalistic paradigm, neural synchrony, intersubject correlation

## Abstract

**Background:**

Autonomous sensory meridian response (ASMR) videos have been increasingly popularized as accessible tools for stress relief. Despite widespread media coverage promoting their benefits, empirical research on the neural mechanisms underlying ASMR remains limited, particularly in general, unselected populations rather than self-identified ASMR responders.

**Objective:**

This study aimed to investigate whether ASMR videos, when viewed in a naturalistic context by a general population sample, elicit consistent neural synchrony in stress-related brain regions and whether individual differences in perceived stress predict variability in neural responses.

**Methods:**

The study included 72 young adults from South Korea. They participated in a functional magnetic resonance imaging experiment in which they viewed 3 ASMR videos selected through both manual and computational content analysis to reflect commonly consumed ASMR content. Intersubject correlation analysis was used to quantify the degree to which participants exhibited shared temporal patterns of neural activity across 16 a priori regions of interest implicated in stress processing. Intersubject representational similarity analysis assessed whether pairwise similarity in self-reported stress levels predicted similarity in neural synchrony. Exploratory analyses examined differences across videos, the impact of familiarity and prior ASMR use, and comparisons with a Mukbang control video.

**Results:**

Intersubject correlation analysis demonstrated that ASMR videos elicited significant neural synchrony in several brain regions (*P*<.05), including the insula and amygdala, although this synchrony varied across videos. No significant associations were identified between perceived stress and neural synchrony after correction for multiple comparisons (all *P*>.05). Exploratory comparisons with a Mukbang control video revealed no significant differences in stress-related neural synchrony between ASMR and non-ASMR content (all *P*>.05). Additional exploratory analyses examining familiarity with the content and prior ASMR viewership also did not show significant effects (all *P*>.05). Uncorrected analyses suggested weak trends that indicated greater neural variability among participants with differing stress levels, but these findings are preliminary.

**Conclusions:**

The results do not provide conclusive evidence that ASMR videos consistently engage stress-related neural networks or that individual stress levels predict neural synchrony during ASMR video viewing. The findings highlight the substantial variability in ASMR engagement across content and individuals, and underscore the need for further research using multimodal physiological measures, larger samples, and stratified designs to identify which ASMR components and viewer characteristics are most relevant to potential stress-relief effects.

## Introduction

The search for effective and affordable stress-relief practices is a critical health issue, given that 41% of adults worldwide report high stress levels, according to a 2021 Gallup survey of 122 countries [1]. The rapid, hectic pace of living, particularly in industrialized societies, exacerbates these stress levels [2], resulting in significant societal costs. Globally, an estimated 12 billion working days are lost each year due to depression and anxiety, which are common stress-related mental health conditions [3]. In the United States alone, work-related stress costs up to US $187 billion annually [4]. The detrimental consequences of stress intensified during the lingering aftermath of the COVID-19 pandemic, which left a noticeable mark on physical and psychological well-being [5].

This study focused on examining an increasingly popular form of online stress-relief practice among young adults in South Korea, a country that reports low life satisfaction, an elevated suicide rate, and alarming levels of mental health issues compared with other industrialized and developed countries [6]. More specifically, it has been reported that higher academic burden and low social support greatly contribute to extreme stress levels and suicidal ideation, especially among younger adults in South Korea [7,8]. Given the strong negative stigma around seeking professional help for mental health challenges [9], many young South Koreans turn to more isolated, implicit forms of stress relief, such as watching videos online.

Among the various types of video content used as a stress-relief strategy, videos that elicit autonomous sensory meridian response (ASMR) are very popular, with 5.2 million ASMR videos on YouTube [10]. ASMR is an involuntary tingling and relaxing sensation that permeates the head, neck, and body [11,12]. Videos that induce ASMR (ie, ASMR videos) often feature a person presenting details of an object in front of the camera and creating repetitive sounds [13]. To fully capture the auditory triggers and enhance the ASMR experience, special binaural microphones are often used to create a sophisticated audioscape [14].

Various theoretical frameworks support the stress-relief effects of digital and online media content. The mood management theory, along with the uses and gratifications theory, suggests that users engage with media to regulate their mood, aligning with hedonic and gratifying goals [15,16]. Therefore, people may select certain media content with the specific intention of reducing stress. Even when experiencing ego depletion, where perseverance in goal attainment is difficult, individuals seek escape through media exposure rather than engaging in other cognitively demanding activities, leading to enjoyment and stress relief [17].

In addition to theoretical arguments, empirical evidence also suggests the potential stress-relief effects of ASMR videos. Researchers and media personalities have recommended ASMR videos as at-home tools for reducing stress, depression, and chronic pain [18-22]. Largely based on self-reported evidence [11,14] and some physiological indicators, such as heart rate, studies have suggested that ASMR videos may reduce stress by promoting relaxation [23,24], providing a meditative experience, mitigating insomnia, improving concentration, and facilitating mental stability [25,26]. Consequently, this has sparked discussions around the clinical application of ASMR videos.

As with any other recommended medical treatment practice, it is important to evaluate the potential of ASMR videos using a combination of self-reports and objective physiological measures. Although self-reports are commonly used to measure stress, they have certain limitations, including susceptibility to subjective biases and variability in individual interpretations [27]. Additionally, self-report measures of perceived stress have low predictive validity for health outcomes [28].

Alternatively, studies have identified more objective measures of stress, such as central nervous system (CNS) indicators [29,30]. When the human brain evaluates the threat posed by a stressor and the resources available for coping, the body typically initiates 2 physiological responses. The first involves the activation of the sympathetic branch of the autonomic nervous system. Specifically, the brainstem and lateral hypothalamus activate sympathetic preganglionic neurons in the spinal cord, which, in turn, stimulate chromaffin cells in the adrenal medulla, prompting the release of epinephrine and norepinephrine into the bloodstream [29]. The second response centers on the activation of the hypothalamic-pituitary-adrenal (HPA) axis. Specifically, corticotropin-releasing hormone and arginine vasopressin, released from the hypothalamus, stimulate the pituitary gland to secrete adrenocorticotropic hormone, which subsequently induces the adrenal cortex to release cortisol [30].

These 2 stress response mechanisms have been empirically corroborated by meta-analyses, which have identified specific brain regions, such as the amygdala, insula, and parahippocampal gyrus, as being integral to stress processing [31,32]. During episodes of stress, corticotropin-releasing hormone is released within the amygdala, a region deeply involved in emotional regulation [33]. The insula, which plays a critical role in perceiving somatic sensations, pain, interoception, and social cognition [34], has been described as part of the paralimbic or limbic integration cortex [35]. Similarly, the parahippocampal gyrus, which is intricately connected to the limbic system, including the amygdala and hippocampus, plays a significant role in regulating the HPA axis. While these regions are central to processing stress, stress-relief stimuli, such as ASMR videos, may exert their effects through overlapping neural pathways [36]. Research on relaxation techniques like meditation and mindfulness suggests that stress-relief practices can reduce activity in stress-processing regions (eg, the amygdala) while influencing the activity in areas linked to emotional regulation and interoception, such as the ventromedial prefrontal cortex, anterior cingulate cortex, and insula [36]. These changes are hypothesized to facilitate a shift from sympathetic activation to parasympathetic nervous system engagement, promoting physiological and psychological relaxation.

Meta-analyses have highlighted other brain regions that, while less directly linked to the autonomic nervous system and HPA axis, are nonetheless involved in stress responses [31,32]. The inferior frontal gyrus, for example, is implicated in semantic and phonological processing, working memory, and fine motor control. Its activation during stress induction paradigms may be attributed to the cognitive demands of tasks such as arithmetic calculations and mental rotations [31]. The globus pallidus, associated with motor preparation and movement, may be involved in the increased muscle tension and preparatory responses characteristic of the fight-or-flight response during stress [31]. Additionally, the superior frontal gyrus, known to activate in conditions of heightened decision uncertainty, may be particularly engaged in stressed individuals [32]. Finally, the putamen, linked to dopamine regulation, and the precuneus, a key node in the brain’s default mode network, have also been implicated in the stress response, though the precise mechanisms of their involvement remain unclear.

Given the popularity of ASMR videos and the growing public interest in their therapeutic potential, it is important to explore the underlying mechanisms that contribute to their psychological effects. Although prior studies suggest that ASMR videos have the potential to reduce stress [23,24], the neurocognitive processes that mediate this effect remain insufficiently explored. While previous neuroimaging research has identified activation in reward- and arousal-related brain regions, such as the nucleus accumbens, insula, and supplementary motor area [37], and altered functional connectivity in the default mode network during resting states [38], these findings primarily reflect data from self-identified ASMR-sensitive individuals under tightly controlled or nonnaturalistic conditions. Consequently, there is a lack of prior research that directly investigates whether ASMR content modulates stress-related neural networks in general, nonscreened populations during naturalistic media exposure.

This gap in the literature further highlights the need to adopt methodological approaches that better reflect how people typically engage with ASMR media. Cognitive neuroscience has traditionally concentrated on simple tasks with abstract stimuli in controlled environments, which has drawn criticism for its limited ecological validity (the extent to which study conditions reflect real-life contexts and behaviors) [39]. Recently, there has been a shift toward naturalistic paradigms, which involve presenting continuous, real-world stimuli, such as films and online videos, to more accurately capture how the brain responds in everyday situations [40]. In neuroimaging research, a naturalistic paradigm enables the investigation of dynamic and temporally complex neural processes, offering richer insights into cognitive and emotional engagement as it unfolds in real time.

Naturalistic paradigms introduce data complexity, requiring advanced analyses to handle diverse stimuli and subject heterogeneity. Traditional subtraction designs, which were commonly employed, may not be well-suited to capture the intricate characteristics of ASMR videos. A subtraction design requires either (1) at least two conditions that differ with respect to the variables of interest while other aspects are the same (if not as similar as possible) or (2) a predefined model based on the variables of interest. For examining the natural experience of watching ASMR videos, these prerequisites remain unmet due to their multidimensional nature and the variance in their content and audiovisual triggers. For instance, while a video recorded using a regular microphone and a specialized binaural microphone can yield 2 stimuli differing in audio quality, they share a common auditory ASMR trigger—whispering and scratching sounds. This challenges the creation of significantly different stimuli as they predominantly vary in only 1 small aspect of ASMR videos. Attempts to introduce further variations would invariably introduce confounding factors. Additionally, constructing a predefined model based on self-report (as done in [41]) or on ASMR triggers would present limitations. The former approach disrupts the natural experience of watching ASMR videos, compelling participants to perform meta-cognitive evaluations of their sensory responses. The latter method is hindered by the fact that ASMR research has yet to definitively identify which specific triggers or combinations are most likely to induce the ASMR experience in which participants and to what degree. This lack of clarity increases the difficulty of creating an effective predefined model.

Considering the inherently naturalistic nature of ASMR videos, this study employed state-of-the-art, subject-based analytical approaches that do not require predefined models and are well-suited for capturing dynamic neural response patterns. Specifically, intersubject correlation (ISC [42]) was used to examine whether watching ASMR videos elicits reproducible, temporally aligned brain responses across participants, thereby quantifying shared neural engagement with the stimuli [41,43]. Building on this foundation, intersubject representational similarity analysis (IS-RSA [44]) was further applied to test whether interindividual differences in perceived stress levels could explain variation in these neural response patterns. In this framework, ISC captures the extent to which ASMR videos evoke consistent brain activity across individuals, while IS-RSA assesses whether participants with similar levels of perceived stress also exhibit more similar neural synchrony profiles. If individuals reporting higher stress levels show greater alignment in stress-related brain regions, this would suggest that ASMR content is processed differently depending on one’s mental state. Conversely, if stress does not account for neural variance, it would indicate that engagement with ASMR videos is influenced by other, potentially idiosyncratic, factors, contributing to the broader discussion of personalized media use for mental health.

## Methods

### Recruitment

The study included 72 young South Korean adults (female: 38/72, 53%; mean age 23.32, SD 3.35 years; age range 20‐30 years). The participants were recruited through an online announcement posted on the official website of Sungkyunkwan University, South Korea. Our sample reflects the population most actively engaging with ASMR globally, with viewership concentrated among young adults aged 18‐32 years in East Asia [11]. Additionally, young South Koreans experience high rates of stress-related mental health issues, making this demographic highly relevant for studying ASMR [45].

### Ethical Considerations

The study received ethical approval from the institutional review boards (IRBs) of Ewha Womans University (IRB approval number: EWHA-201905-0021-01) and Sungkyunkwan University (IRB approval number: SKKU 2015-11-012). All participants provided written informed consent in Korean prior to participation. The consent procedure specified that participants would be exposed to a range of video stimuli, including both ASMR and non-ASMR content, and that their responses could be compared across these conditions. Participants received a gift certificate valued at 50,000 won (approximately US $45 at the time of the study) as compensation for their participation. All data were anonymized prior to analysis, and no personally identifiable information was retained.

### Behavioral Measures

Before undergoing magnetic resonance imaging (MRI), participants completed a survey assessing their stress levels. The survey comprised 6 items on a 5-point Likert scale ranging from 1 (“never experienced”) to 5 (“always”) [46], which was translated into Korean [47] (see Multimedia Appendix 1 for the full scale). The survey included items such as “In the past month, how often have you been upset because of something that happened unexpectedly?” and “In the past month, how often have you felt that you were unable to control the important things in your life?” A higher score indicates a higher stress level.

To further understand participants’ experiences and attitudes toward ASMR content, a postexperiment questionnaire was administered following the MRI session. Specifically, participants reported (1) how frequently they watched ASMR videos, rated on a 5-point Likert scale ranging from 1 (never) to 5 (very often); (2) whether they were familiar with each of the 3 YouTube channels from which the ASMR video stimuli were drawn (yes/no); and (3) whether they would be willing to continue watching ASMR videos in the future (yes/no).

### Functional MRI Acquisition and Preprocessing

Participants were screened for MRI contraindications before scanning at a Korean brain imaging institution (Sungkyunkwan University’s N-Center) using a Siemens 3T Magnetom Prisma scanner running IDEA software version VE11C (Siemens Healthineers). T1-weighted structural MRI data were acquired using the magnetization-prepared rapid gradient echo (MPRAGE) sequence (slices=320, isotropic voxels=0.7 mm, repetition time=2400 ms, echo time=2.34 ms, field of view=224×224 mm). Functional magnetic resonance imaging (fMRI) data were obtained through the gradient-echo echo-planar imaging sequence (volumes=1728, slices=72, isotropic voxels=2.0 mm, repetition time=1000 ms, echo time=39.80 ms, field of view=224×224 mm). The MPRAGE sequence lasted approximately 7 minutes and 20 seconds, and the echo-planar imaging sequence lasted approximately 29 minutes. The total MRI session, including participant setup, localizer scans, anatomical and functional acquisitions, and brief rest breaks, was approximately 45‐50 minutes per participant. Acquisition parameters were selected to balance high spatial and temporal resolution with full-brain coverage for modeling dynamic audiovisual stimuli and intersubject neural synchrony [42,48], and followed standard MPRAGE parameters recommended for high-resolution anatomical imaging [49]. Additionally, the protocol was optimized to meet the capabilities and technical specifications of the imaging facility at Sungkyunkwan University.

Each participant’s fMRI scanning session consisted of a continuous run featuring 3 ASMR videos presented in a random order (videos are available on Open Science Framework [50] and in Multimedia Appendices 2-4). The stimuli selection process involved manual content analysis and computational analysis. Initially, popular channels and viewership metrics guided video selection. Subsequently, manual content analysis ensured the inclusion of essential ASMR audiovisual features. Finally, computational analysis of low-level audiovisual features, including amplitude and frequency for audio, and hue, saturation, and value for visual, was conducted to ensure that these features were not extraordinary (more details are available in Multimedia Appendix 5). These video stimuli were back-projected onto a screen positioned at the end of the scanner bore and viewed by participants via a mirror mounted on the radiofrequency coil. The audio was delivered through MRI-compatible headphones equipped with noise-canceling capabilities.

The fMRI data were preprocessed using fMRIPrep 20.2.1 (fMRIPrep Development Team), which encompassed motion correction (MCFLIRT), nonbrain removal (BET), and spatial smoothing with a Gaussian kernel of full width at half maximum of 3.0 mm [51]. Additionally, multiplicative mean intensity normalization of the volume at each time point and high-pass temporal filtering (Gaussian-weighted least-squares straight-line fitting with sigma=55.5 s) were applied [52].

**Figure 1.**
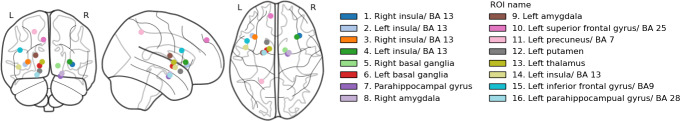
Glass brain visualization of selected regions of interest (ROIs) associated with stress. Sixteen brain regions are represented using differently colored dots. BA: Brodmann area.

As stress-associated CNS indicators were of interest, 16 spherical regions of interest (ROIs) with a 6-mm diameter were selected based on the Montreal Neurological Institute (MNI) standard coordinates provided by previous meta-analyses showing convergence in brain activity for stress processing [31,32]. These include the bilateral insula, bilateral basal ganglia, bilateral amygdala, left superior frontal gyrus, left inferior frontal gyrus, left precuneus, left putamen, left thalamus, and left parahippocampal gyrus (Figure 1).

### Statistical Analysis

We employed subject-based analyses to investigate the synchrony of neural responses within and across subjects using both ISC analysis and the IS-RSA framework. ISC relies on the premise that participants exhibiting similar neural patterns while processing identical audiovisual information likely share common perceptual and cognitive processes. In other words, ISC analysis highlights localized spatial processing by filtering out subject-specific signals and revealing ROIs with consistent, stimulus-evoked response time series across subjects [53].

First, to assess whether neural synchrony differed across the 3 ASMR videos, ISC was computed separately for each video by calculating the pairwise Pearson correlation coefficients between every possible pair of participant time series. The resulting ISC values were Fisher *z*-transformed to improve normality. A Friedman test was then conducted within each ROI to evaluate omnibus differences in ISC across the 3 videos. For ROIs where the Friedman test indicated a significant effect, post hoc Wilcoxon signed-rank tests were performed to compare ISC between video pairs. For all analyses, including the subsequent ones, *P *values were corrected for multiple comparisons across ROIs using false discovery rate (FDR) procedures.

To evaluate the overall consistency of neural responses irrespective of video content, ISC was also computed on neural time series concatenated across all 3 ASMR videos. This approach yielded a single ISC estimate per ROI that captured shared temporal dynamics across the entire experimental session. For all ISC analyses, statistical significance was assessed using subject-wise bootstrapping with 5000 iterations.

Second, if watching ASMR videos is an effective stress-relief practice, then highly stressed individuals should engage with and process these videos in more similar ways than low-stressed individuals, particularly in brain regions associated with stress relief. To test this, we used IS-RSA to examine the relationship between the similarity in neural responses and the similarity in stress levels. This approach aligns with the analytical framework used in previous studies that have demonstrated the utility of IS-RSA for linking shared neural representations to shared psychological traits or states [44]. Spearman ρ rank correlations assessed the correlation between 2 matrices: the pairwise similarity matrix of neural responses (constructed using ISC analysis for ASMR videos) and the pairwise similarity matrix of stress levels. The latter matrix was generated using a similarity metric according to the Anna Karenina model [44], which leads to high similarities in stressed individuals who exhibit consistent and shared neural responses when exposed to the same stimuli but low similarities in individuals who are not stressed and do not share a unified stress-processing pattern. Following the same procedure applied in the ISC analysis, IS-RSA was conducted separately for each ASMR video, and differences in associations across videos were evaluated using Friedman tests. Additionally, IS-RSA was performed on neural time series concatenated across all ASMR videos to estimate the overall relationship between stress similarity and neural similarity across the entire ASMR viewing session.

The ISC analysis and IS-RSA examining associations with perceived stress are directly related to the study’s primary goal: to evaluate the validity of the general claim that watching ASMR videos may function as an effective stress-relief practice. To further understand the neural responses associated with the ASMR video viewing experience beyond this goal, we conducted several exploratory analyses that were not preregistered in the original protocol. These analyses are reported separately to distinguish them from the primary hypothesis tests.

To further investigate whether neural representational similarity during ASMR video viewing reflected individual differences in perceived stress, we conducted an exploratory comparison with a naturalistic non-ASMR control stimulus. Specifically, we selected a Mukbang video, which is a popular genre featuring individuals consuming large quantities of food, as the comparison condition. Mukbang videos are comparable to ASMR content in their online popularity and solitary viewing context but lack the characteristic auditory enhancements typical of ASMR. IS-RSA was performed for the Mukbang video following the same procedures used for the ASMR videos. To evaluate whether stress-related neural similarity was systematically higher during ASMR video viewing than during Mukbang video viewing, each ASMR video was paired with the Mukbang video, and IS-RSA scores across ROIs were compared using paired-samples *t* tests.

We also explored whether the ASMR viewing experience modulated intersubject neural synchrony. Participants were grouped based on their dichotomous responses regarding (1) willingness to continue watching ASMR videos and (2) familiarity with each ASMR channel. ISC was computed separately for each group, and differences in ISC values across groups were evaluated using Friedman tests. Finally, to assess whether similarity in ASMR viewing frequency predicted similarity in neural responses, we conducted an additional IS-RSA using a behavioral similarity matrix that was constructed by calculating pairwise similarity scores based on participants’ self-reported ASMR viewing frequency for each ROI.

## Results

### Behavioral Measures

The 6 items measuring perceived stress demonstrated good reliability (Cronbach *α*=0.80). A composite variable was created by calculating the average, which indicated moderate perceived stress levels among the participants, with a mean stress score of 2.33 (SD 0.66; range 1.17‐3.83). Regarding prior viewing frequency, the mean self-reported frequency was relatively low (mean 1.56, SD 1.15) on a 5-point Likert scale ranging from 1 (never) to 5 (very often). Familiarity with the YouTube channels from which the videos were drawn varied: 71% (46/65) of participants reported having previously encountered the channel for video 1 (Multimedia Appendix 2), 11% (7/65) were familiar with the channel for video 2 (Multimedia Appendix 3), and 5% (3/65) were familiar with the channel for video 3 (Multimedia Appendix 4). There was no statistically significant relationship between the behavioral measures (all *P*>.05).

### ISC Findings

To evaluate whether neural synchrony differed across the 3 ASMR videos, we computed ISC separately for each video and used the Friedman test for comparisons. The results demonstrated that 5 ROIs showed significant differences across videos: the right parahippocampal gyrus (*χ*^2^_2_=19.53; *P*<.001, FDR corrected), right amygdala (*χ*^2^_2_=16.36; *P*=.002, FDR corrected), left precuneus (*χ*^2^_2_=13.53; *P*=.006, FDR corrected), left inferior frontal gyrus (*χ*^2^_2_=11.44; *P*=.01, FDR corrected), and left insula (*χ*^2^_2_=10.58; *P*=.047, FDR corrected). Post hoc Wilcoxon signed-rank comparisons indicated that ISC during video 3 (carving video) was significantly higher than that during the other videos in the right amygdala and left inferior frontal gyrus (minimum *W*=863; *z*=2.53; *P*=.01, FDR corrected). In the right parahippocampal gyrus and left precuneus, ISC during video 3 was significantly higher than that during video 1 (minimum *W*=881; *z*=2.43; *P*=.04, FDR corrected). In the left insula, ISC during video 3 was significantly higher than that during video 2 (*W*=866; *z*=2.51; *P*=.047, FDR corrected).

**Figure 2.**
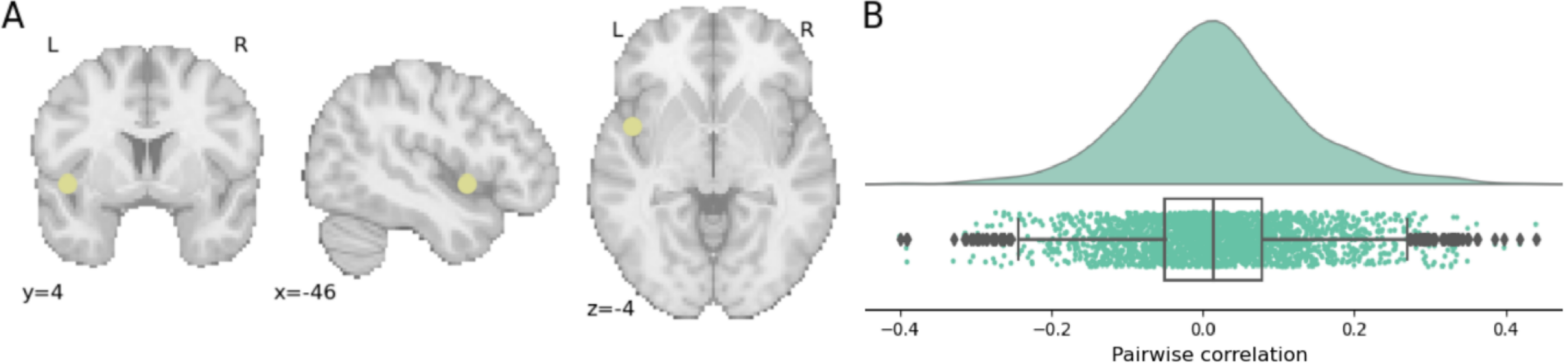
Distribution of pairwise correlations between subjects for the left insula. (A) Montreal Neurological Institute coordinates of the left insula. (B) Range and distribution of neural synchrony (intersubject correlation values) across all participant pairs in the left insula, visualized through a histogram, box plot, and scatter plot.

When ISC across all 3 videos was examined using concatenated time series, the ISC analysis revealed significant neural synchrony in the left insula (MNI coordinates: −46, 4, −4; ISC score=0.0134; *P*=.003, FDR corrected). This finding indicates a consistent neural response among participants while viewing ASMR videos in this region. The left insula is a key region involved in interoceptive awareness and emotional processing, suggesting that its activity may reflect a shared experience elicited by ASMR stimuli. Figure 2 illustrates the distribution of pairwise correlations between participants for the left insula, showing the range of synchrony across the sample.

### IS-RSA Findings

The IS-RSA examined the relationship between neural synchrony and participants’ perceived stress levels. When IS-RSA was conducted separately for each ASMR video, no significant associations were observed in any ROI (all *P*>.05), even before applying FDR correction. To evaluate whether stress modulated neural synchrony across the entire ASMR viewing session, IS-RSA was also performed on the neural time series concatenated across all 3 videos. After applying FDR correction, no statistically significant relationships were identified (all *P*>.05). However, exploratory analysis of uncorrected results indicated negative correlations in 2 regions: the right insula (IS-RSA score=−0.0606) and right amygdala (IS-RSA score=−0.0477). These results suggested a trend where greater differences in stress levels between participant pairs were associated with greater variability in neural responses to ASMR videos in these regions. While the uncorrected findings did not meet the threshold for statistical significance, they highlight potential patterns that warrant further investigation. Table 1 provides a summary of ISC and IS-RSA findings.

**Table 1.** Summary of intersubject correlation and intersubject representational similarity analysis findings.

Region of interest^a^	MNI^b^ coordinates	ISC^c^^,^^d^ results	IS-RSA^e^^,f^ results
Right insula/BA^g^ 13	[36, 22, 0]	0.0049	−0.0606^h^
Left insula/BA 13	[−36, 22, 0]	0.0070	−0.0088
Right insula/BA 13	[−32, 20, 4]	0.0071	−0.0442
Left insula/BA 13	[32, 20, 4]	0.0021	−0.0239
Right basal ganglia	[14, 0, −2]	0.0052	−0.0052
Left basal ganglia	[−14, 0, −2]	0.0003	0.0017
Right parahippocampal gyrus	[18, −6, −18]	0.0043	0.0031
Right amygdala	[20, −4, −14]	0.0043	−0.0477^h^
Left amygdala	[−20, −4, 14]	0.0038	0.0029
Left superior frontal gyrus/BA 25	[−8, 52, 38]	0.0134	−0.0117
Left precuneus/BA 7	[−22, −48, 50]	0.0045	−0.0369
Left putamen	[−14, 12, −10]	0.0003	0.0006
Left thalamus	[−10, 2, 4]	0.0006	−0.0142
Left insula/BA 13	[−46, 4, −4]	0.0134^h^^,^^i^	−0.0032
Left inferior frontal gyrus/BA 9	[−44, 14, 22]	0.0084	−0.0584
Left parahippocampal gyrus/BA 28	[−18, −4, −16]	−0.0040	−0.0051

a The regions of interest are indicated in Figure 1.

b MNI: Montreal Neurological Institute.

c ISC: intersubject correlation.

d Pearson correlation is shown.

e IS-RSA: intersubject representational similarity analysis.

f Spearman correlation is shown.

g BA: Brodmann area.

h *P*<.05 (uncorrected).

i Statistical significance after false discovery rate correction.

### Exploratory Analysis

To further investigate whether neural synchrony occurs in other brain regions, we conducted further ISC analyses using 323 nonoverlapping ROIs, each with a 6-mm spherical volume, and clustered the ROIs based on anatomical approximation. These ROIs were selected based on association test maps from Neurosynth’s meta-analytic co-activation of 190 studies that included the term “video” [54]. The association test maps assessed the consistency of activation in specific regions for studies mentioning “video” compared to those that did not. As shown in Table 2, participants exhibited neural synchrony in brain regions associated with visual processing, auditory processing, sensory-motor functions, and areas involved in higher cognitive processing. This indicates that ASMR videos elicit similar neural processing patterns across participants watching the given set of videos, primarily in lower sensory brain regions.

**Table 2.** Intersubject correlation results of exploratory analysis on regions of interest related to “video.”

Brain regions	Number of ROIs^a^	ISC-Max^b^^,^^c^	*P* value	MNI^d^ coordinates^e^		
Visual processing regions						
Intracalcarine cortex	8	0.105	<.001	[−4, −84, 2]		
Lateral occipital cortex, inferior division	7	0.051	<.001	[−48, −72, 6]		
Lateral occipital cortex, superior division	10	0.035	<.001	[17, −60, 68]		
Lingual gyrus	8	0.091	<.001	[−6, −81, −4]		
Occipital fusiform gyrus	3	0.054	<.001	[24, −90, −8]		
Occipital pole	12	0.087	<.001	[8, −95, 19]		
Auditory processing regions						
Planum temporale	2	0.026	.005	[−57, −26, 13]		
Superior temporal gyrus, posterior division	2	0.077	<.001	[58, −28, 8]		
Sensory-motor regions						
Postcentral gyrus	2	0.057	<.001	[−66, −20, 32]		
Precentral gyrus	1	0.026	.01	[−56, 10, 28]		
Supramarginal gyrus, anterior division	1	0.022	<.001	[−57, −31, 34]		
Supramarginal gyrus, posterior division	1	0.010	.02	[46, −38, 14]		
Higher cognition regions						
Inferior temporal gyrus, posterior division	1	0.025	<.001	[54, −10, −42]		
Middle temporal gyrus, temporo-occipital part	2	0.057	<.001	[50, −44, 4]		
Precuneous cortex	1	0.014	.02	[−12, −70, 30]		
Superior parietal lobule	10	0.058	<.001	[30, −52, 65]		
Temporal occipital fusiform cortex	4	0.043	<.001	[−30, −60, −12]		
Temporal pole	1	0.068	<.001	[59, 12, −2]		

a ROIs: regions of interest.

b ISC: intersubject correlation.

c ISC-Max: the highest Pearson correlation value among the regions of interest with anatomical approximation.

d MNI: Montreal Neurological Institute.

e MNI coordinates of the region of interest with the highest intersubject correlation are reported.

**Figure 3.**
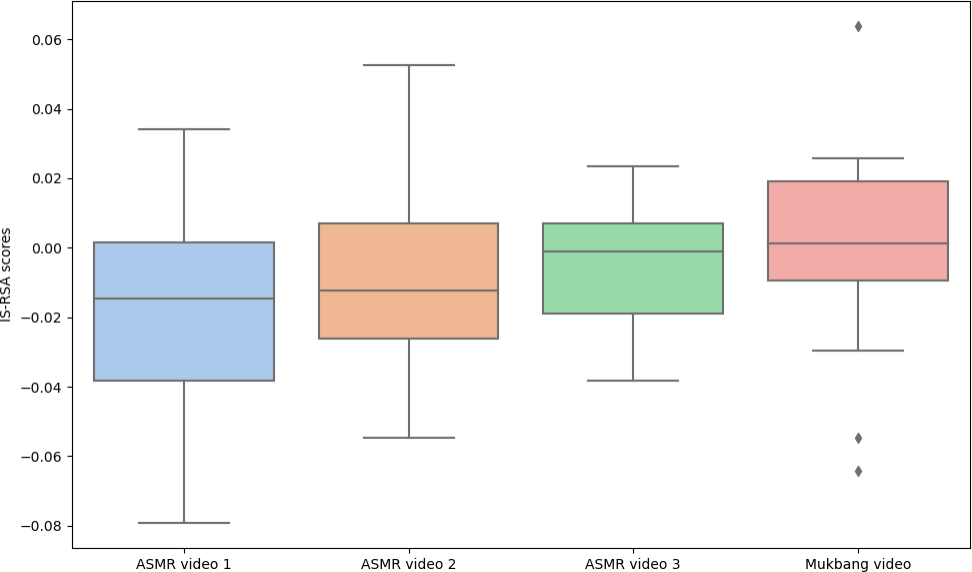
Box plots of intersubject representational similarity analysis (IS-RSA) scores for 3 autonomous sensory meridian response (ASMR) videos and 1 Mukbang video used as a naturalistic control. Each box represents the distribution of IS-RSA values across participant pairs within each condition. The central line denotes the median, the box represents the IQR, and the whiskers extend to 1.5 times the IQR. No significant difference was observed between the Mukbang video and ASMR video 1 (*P*=.08), ASMR video 2 (*P*=.35), or ASMR video 3 (*P*=.60).

To further investigate whether neural representational similarity in response to ASMR videos reflects individual differences in stress levels, we conducted an additional analysis using a comparative stimulus, a Mukbang video. The results revealed no statistically significant differences in neural representational similarity (*t*_15_=0.54; *P*=.60 to *t*_15_=1.90; *P*=.08). Figure 3 illustrates a box plot of the IS-RSA scores for each of the 3 ASMR videos and the Mukbang video. Therefore, it is inconclusive to state that the degree to which neural responses vary as a function of stress is amplified during ASMR video viewing.

To assess whether prior experience with ASMR content influenced neural synchrony, we conducted exploratory analyses relating individual viewing habits and familiarity to ISC and representational similarity. Comparisons of ISC between participants who were willing versus unwilling to continue watching ASMR videos revealed no significant group differences across any ROI (all *P*>.05 after FDR correction). Similarly, analyses comparing ISC by prior familiarity with each ASMR channel yielded no significant effects across ROIs or videos (all *P*>.05). Notably, in some cases (eg, familiarity with channel 3 endorsed by only 3 participants), limited sample sizes precluded reliable inferences. Finally, IS-RSA tested whether the self-reported frequency of ASMR video viewing predicted neural response similarity across participants. However, no significant associations were observed in any ROI (all *P*>.05 after FDR correction).

## Discussion

### Principal Findings

This study examined whether ASMR videos elicit consistent neural responses across individuals and whether these responses are modulated by perceived stress levels. The findings indicated that the carving video, which prominently featured repetitive scraping sounds and a focused visual target, elicited significantly stronger intersubject neural synchrony than the other 2 ASMR videos. This suggests that the specific characteristics of ASMR content may shape the degree of shared neural engagement and underscores the importance of considering the variability of ASMR video content.

Consistent with prior research, ISC analysis revealed significant synchrony in the left insula, a region implicated in interoception, emotional processing, and the regulation of autonomic nervous system activity [37]. This pattern aligns with the notion that ASMR experiences engage brain regions supporting bodily awareness and affective responses. However, these results should be interpreted with caution and considered preliminary, given the novel application of ISC to ASMR video analysis, as well as other methodological aspects of this study discussed below. Replication in independent samples will be important to determine the robustness and generalizability of these findings.

Likewise, IS-RSA did not identify significant associations between neural synchrony and self-reported stress levels. Exploratory, uncorrected analyses suggested a weak trend toward negative correlations in the right insula and right amygdala, which could indicate that higher stress levels are associated with greater variability in neural responses to ASMR videos. However, because these effects were not confirmed by statistical correction, they should be interpreted cautiously and considered preliminary.

An important consideration in interpreting these findings is the absence of screening for ASMR sensitivity. Unlike prior neuroimaging studies that exclusively recruited ASMR-sensitive individuals [37,38], this study intentionally included an unselected sample to enhance ecological validity and better reflect general population engagement with ASMR content. While this approach increases generalizability, it likely introduces additional variability in participants’ responsiveness to ASMR stimuli, potentially reducing statistical power to detect subtle effects in stress-related neural circuits. Accordingly, the lack of robust associations between perceived stress and neural synchrony does not provide definitive evidence against the potential stress-relief effects of ASMR videos but instead highlights the need for further research in both screened and unscreened samples.

### Comparison With Prior Work

The findings of this study differ from those of prior studies that have reported the stress-reducing effects of ASMR videos, particularly those based on self-reported outcomes [11,14] or peripheral physiological markers such as heart rate and blood pressure [24,55,56]. These studies have demonstrated reductions in heart rate, lowered blood pressure, or improvements in mood following exposure to ASMR videos. However, most of this prior work has focused on autonomic indicators or self-identified ASMR responders, rather than investigating CNS mechanisms within unstratified populations.

While peripheral nervous system measures are valuable for detecting immediate physiological changes, they do not directly reflect the neural substrates of stress processing. In contrast, this study used fMRI-based methods (ISC and IS-RSA) to examine how individual differences in perceived stress relate to neural engagement during ASMR viewing. This approach was designed to assess whether ASMR videos elicit distinct patterns of neural processing in individuals with higher stress levels, which would be expected if ASMR videos function as a targeted stress-relief intervention.

Given our large sample size and the use of carefully curated ASMR stimuli selected through both manual and computational methods [57], the absence of robust stress-related neural effects suggests a need for more precision in how ASMR is studied and discussed. The discrepancy between our findings and those of earlier studies may be attributed to methodological differences, including measurement modality and population sampling [23,37]. Prior studies frequently recruited participants who self-identified as ASMR responders or who reported experiencing ASMR tingles [23,37], whereas our study included a general population sample without stratification based on ASMR sensitivity. This distinction is essential, as benefits from ASMR may be more pronounced among individuals predisposed to ASMR responsiveness [14,23,24]. Prior studies that did not account for ASMR responsiveness often reported null or mixed outcomes, and some studies found no significant changes in mood or even reductions in positive affect among nonresponders exposed to ASMR videos [58,59]. These findings suggest that ASMR may function as a therapeutic tool primarily for those who are sensitive to it, and caution should be exercised when making generalized claims about its effectiveness in stress reduction for broader populations.

Additionally, prior research has described ASMR as producing a complex blend of affective states, including both relaxation and arousal [26,60]. This duality complicates the simplified narrative of ASMR as a uniformly stress-reducing experience. The lack of consistent effects in stress-related brain regions observed in our study may reflect this complexity, as well as the possibility that ASMR’s sensory and emotional effects engage general-purpose affective systems rather than stress-specific pathways.

### Limitations and Future Work

This study sought to maximize ecological validity by presenting popular ASMR videos in a naturalistic manner and recruiting a general population sample, rather than preselecting individuals who self-identify as highly sensitive to ASMR experiences. While this approach improves the relevance of the findings for evaluating broad claims about ASMR’s potential stress-relief benefits, it also introduces several important limitations.

First, the sample consisted exclusively of young South Korean adults. This demographic was intentionally selected due to the high prevalence of stress-related mental health concerns among young people in South Korea, as well as the cultural prominence of ASMR content on Korean media platforms. South Korea’s media ecology, which is characterized by high engagement with personalized content, strong parasocial interaction norms, and widespread use of ASMR for concentration and emotional self-regulation, may shape how ASMR is cognitively and affectively processed. Additionally, cultural factors related to stress perception and emotional expression could influence the degree to which ASMR content engages stress-related brain systems. Therefore, future research may extend these findings by including broader age ranges or cross-cultural comparisons to examine the universality of neural mechanisms underlying ASMR.

Second, although the 3 ASMR videos were carefully selected through manual and computational content analysis, they still exhibited considerable variability in their audiovisual characteristics [61], which may have influenced their subjective appeal. Exploratory analyses indicated that 1 video (soap carving) elicited higher neural synchrony than the others, suggesting that different ASMR stimuli engage participants to different extents. However, we did not collect self-reported ratings of how appealing or enjoyable participants found each video. This is an important limitation, as subjective evaluations could provide critical insights into the variability of neural responses and help clarify whether stronger synchrony reflects shared positive affect, attentional engagement, or other factors unrelated to stress reduction. Collecting such ratings in future studies could help contextualize individual differences in engagement and clarify how perceived appeal relates to brain responses. To balance experimental control with ecological validity, future research may also consider designs that systematically vary specific ASMR components (eg, sound type and visual focus) and measure postexperiment self-reported ratings on these components while preserving the naturalistic presentation format.

Third, while the sample size of 72 participants is relatively large compared with that in many fMRI studies using naturalistic paradigms [39,57], it is possible that the statistical power was insufficient to detect small or heterogeneous effects of ASMR and that the analytic approach, while state-of-the-art, was less sensitive to subtle or idiosyncratic neural patterns across participants. This limitation is especially relevant given the absence of statistically significant relationships between perceived stress and neural synchrony after correction for multiple comparisons. Accordingly, these null findings should be interpreted with caution and viewed as preliminary rather than definitive evidence against ASMR’s stress-relief potential. Future studies using larger samples with stronger inducement of ASMR could more robustly evaluate these associations.

Fourth, this study relied on fMRI-based measures and self-reports on stress without integrating peripheral physiological indicators of stress, such as heart rate variability, skin conductance, and cortisol levels. While this is a common limitation in ASMR research, where most studies rely solely on self-report measures, it nonetheless restricts the construct validity of stress assessment. Given that stress involves both CNS and autonomic nervous system responses, future research should incorporate multimodal physiological assessments to obtain a more comprehensive picture of the mechanism of ASMR videos and their relation to stress levels.

Finally, although the inclusion of a Mukbang video as a naturalistic control provided useful context for interpreting neural synchrony, this approach cannot fully disentangle the effects of ASMR content from other factors, such as attentional engagement. Future research could benefit from comparisons with established stress-relief interventions, such as meditation [40], to better examine the potential stress-relief mechanism. Together, these refinements may help identify the specific mechanisms and user populations for which ASMR may be most effective.

### Conclusion

This study examined whether ASMR videos, when viewed in a naturalistic context by a general population sample, elicit consistent neural synchrony in stress-related brain regions and whether individual differences in perceived stress predict variability in neural responses. Although the naturalistic paradigm offers valuable ecological validity, the results did not identify significant associations between perceived stress levels and intersubject neural similarity. Instead, the analyses revealed variation across different ASMR videos. Overall, the findings do not provide conclusive evidence that ASMR videos consistently modulate stress-related neural networks among unselected viewers. These results underscore the importance of continued research before making broad claims that ASMR videos “work for stress relief,” as is often implied in popular media coverage (eg, “How ASMR is Helping People Improve Their Mental Health”) [62]. In light of this and previous studies, it appears more accurate to suggest that *certain* ASMR videos may be effective for *certain* individuals, rather than assuming universal benefits. At present, the understanding of who responds to ASMR content, which features are most effective, and under what conditions these effects occur remains limited.

## Supplementary material

10.2196/68586 Multimedia Appendix 1 Perceived Stress Scale used in the participant survey.

10.2196/68586 Multimedia Appendix 2 Autonomous sensory meridian response water video stimulus (3-minute segment).

10.2196/68586 Multimedia Appendix 3 Autonomous sensory meridian response carving video stimulus (3-minute segment).

10.2196/68586 Multimedia Appendix 4 Autonomous sensory meridian response eating video stimulus (3-minute segment).

10.2196/68586 Multimedia Appendix 5 Description and validation of the autonomous sensory meridian response video stimuli.

## Abbreviations

ASMR: autonomous sensory meridian response
CNS: central nervous system
FDR: false discovery rate
fMRI: functional magnetic resonance imaging
HPA: hypothalamic-pituitary-adrenal
IRB: institutional review board
IS-RSA: intersubject representational similarity analysis
ISC: intersubject correlation
MNI: Montreal Neurological Institute
MPRAGE: magnetization-prepared rapid gradient echo
MRI: magnetic resonance imaging
ROI: region of interest
