# Supplementary material for: Exploring Neural Idiosyncrasies in Response to Autonomous Sensory Meridian Response Videos: Naturalistic Functional Magnetic Resonance Imaging Study of Stress and Sensory Processing
Source: J Med Internet Res. 2025 Jul 29;27:e68586. doi: 10.2196/68586 (PMC12306919; doi:10.2196/68586)
Supplement: Multimedia Appendix 1 [file jmir-v27-e68586-s001.docx]

**Multimedia Appendix 1**

Perceived stress was assessed using items from the Korean-translated and validated version of the Perceived Stress Scale (PSS) originally developed by Cohen et al. (1983) and translated by Park and Seo (2010). The PSS is a widely used instrument for measuring subjective stress and has been adapted into multiple languages (e.g., Japanese: Mimura et al., 2008; Arabic: Almadi et al., 2012). In Korea, the PSS has demonstrated validity across age groups, including adolescents (Yoon & Kim, 2019) and older adults (Hwang & Jeon, 2020).

For this study, six items were selected to shorten the administration time. This shorter version has also been shown to be suitable for non-clinical samples (Henry & Crawford, 2005).

Items were rated on a 5-point Likert scale ranging from 1 = Never Experienced to 5 = Always.

Original Korean Items

1. 지난 한 달 동안, 예상치 못한 일 때문에 화난 적이 있으십니까?
2. 지난 한 달 동안, 인생의 중요한 것을 스스로 통제하지 못한다고 느끼신 적이 있으십니까?
3. 지난 한 달 동안, 긴장되거나 스트레스 받은 적이 있으십니까?
4. 지난 한 달 동안, 일이 자신이 원하는 대로 풀린다고 느끼신 적이 있으십니까? *
5. 지난 한 달 동안, 해야 할 일을 모두 감당할 수 없다고 느끼신 적이 있으십니까?
6. 지난 한 달 동안, 어려움이 너무 쌓여 해결할 수 없다고 느끼신 적이 있으십니까?

(*) Reverse-coded item

English Back-Translation of the Items

1. In the past month, how often have you been upset because of something that happened unexpectedly?
2. In the past month, how often have you felt that you were unable to control the important things in your life?
3. In the past month, how often have you felt nervous or stressed?
4. In the past month, how often have you felt that things were going your way? (*)
5. In the past month, how often have you felt that you could not cope with all the things you had to do?
6. In the past month, how often have you felt that difficulties were piling up so high that you could not overcome them?

(*) Reverse-coded item

**References**

Arnold, S. V., Smolderen, K. G., Buchanan, D. M., Li, Y., & Spertus, J. A. (2012). Perceived stress in myocardial infarction: Long-term mortality and health status outcomes. *Journals of the American College of Cardiology, 60*(18), 1956-1763. https://doi.org/10.1016/j.jacc.2012.06.044

Cohen, S., Kamarck, T., & Mermelstein, R. (1983). A global measure of perceived stress. *Journal of Health and Social Behavior, 24*(4), 385–396. https://doi.org/10.2307/2136404

Creswell, D., Brown, K. W., Cohen, S., Creswell, K., Zoccola, P., Dickerson, S., Dutcher, J., Wu, S., & Chin, B. (2025). Does high perceived stress over the past month alter cortisol reactivity to the trier social stress test? *Phychoneuroendocrinology, 172*, 107256. https://doi.org/10.1016/j.psyneuen.2024.107256

Henry, J. D., & Crawford, J. R. (2005). The short-form version of the Depression Anxiety Stress Scales (DASS-21): Construct validity and normative data in a large non-clinical sample. *British Journal of Clinical Psychology, 44*(2), 227-239.<https://doi.org/10.1348/014466505X29657>

Lindholdt, L., Labriola, M., Adnersen, J. H., Kjeldsen, M. Z., Obel, C., & Lund, T. (2022). Perceived stress among adolescents as a marker for future mental disorders: A prospective cohort study. *Scandinavian Journal of Pubic Health, 50*, 412-417. https://doi.org/10.1177/1403494821993719

Mimura, C., & Griffiths, P. A (2008) Japanese version of the Perceived Stress Scale: cross-cultural translation and equivalence assessment. *BMC Psychiatry 8*, 85.
