# Supplementary material for: Exploring Neural Idiosyncrasies in Response to Autonomous Sensory Meridian Response Videos: Naturalistic Functional Magnetic Resonance Imaging Study of Stress and Sensory Processing
Source: J Med Internet Res. 2025 Jul 29;27:e68586. doi: 10.2196/68586 (PMC12306919; doi:10.2196/68586)
Supplement: Multimedia Appendix 5 [file jmir-v27-e68586-s005.docx]

**Multimedia Appendix 5**

This study utilized three ASMR videos. The first video^^[[1]](#footnote-0)^^ showcases a person eating deep-fried chicken, referred to as the "eating video" for simplicity. The second video^^[[2]](#footnote-1)^^ involves a person performing slow and repetitive motions with water, which we will call the "water video." This includes dripping water into a bowl, blowing air into a water-filled glass bottle, pouring water from a cup to a bowl, and shaking a water-filled glass bottle. The third video^^[[3]](#footnote-2)^^ features a person carving a bar of soap, referred to as the "carving video." All the videos were popular, with 227 million, 443 million, and 1923 million views, respectively, at the time of the study. Screenshots of the videos are shown below.

**Figure 1**. **Screenshots of the Stimuli**

| A  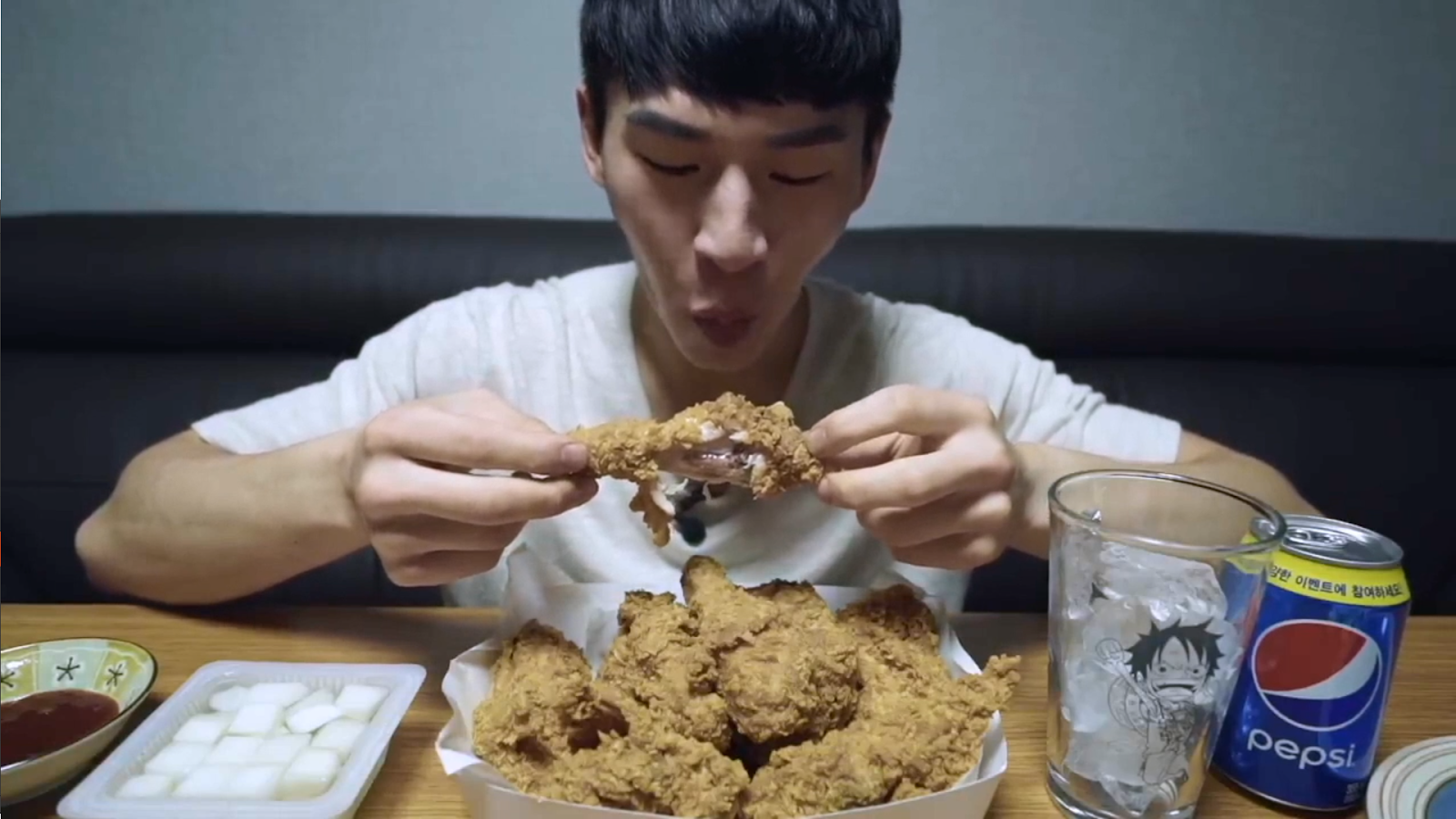 | B  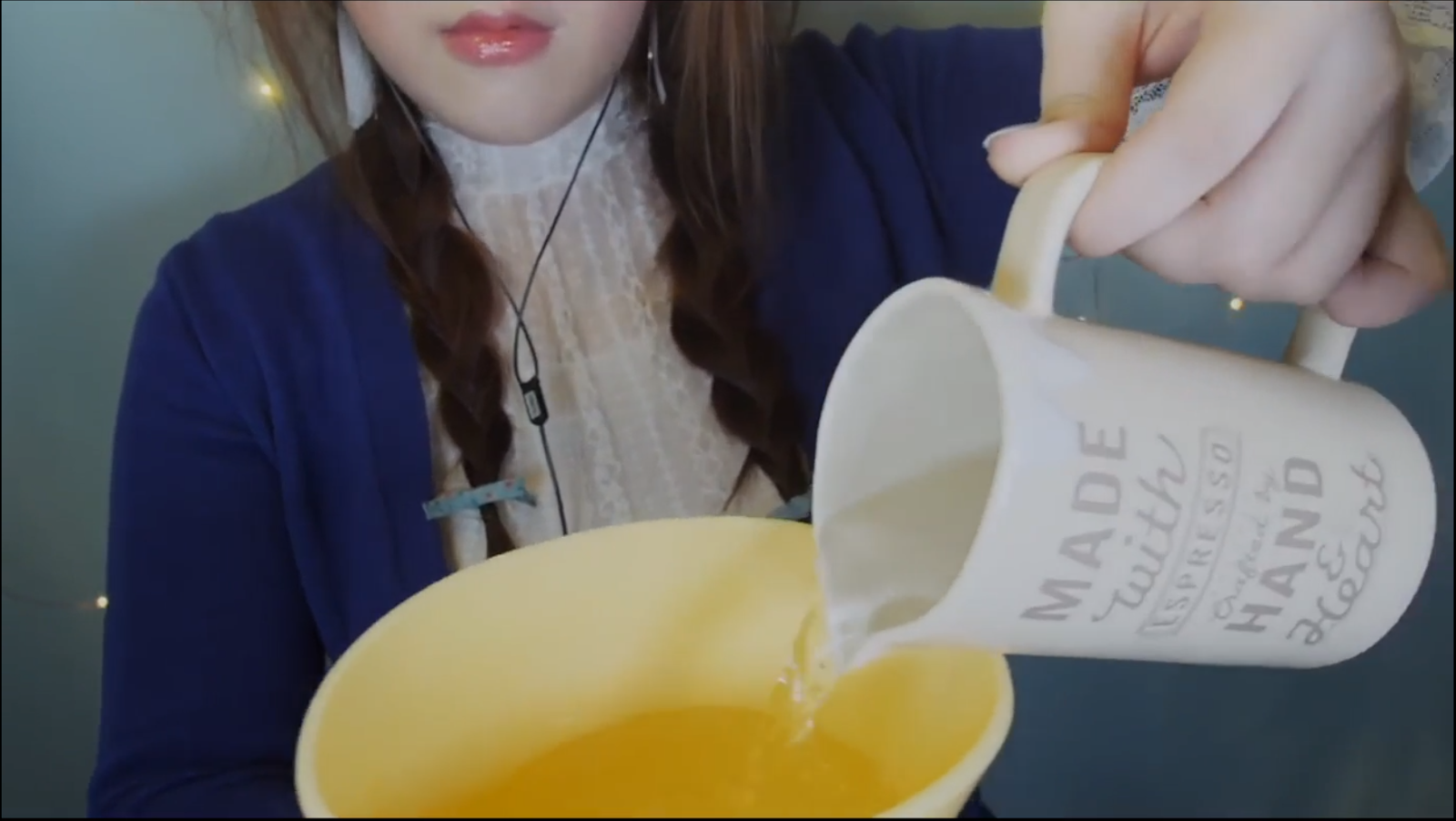 | C  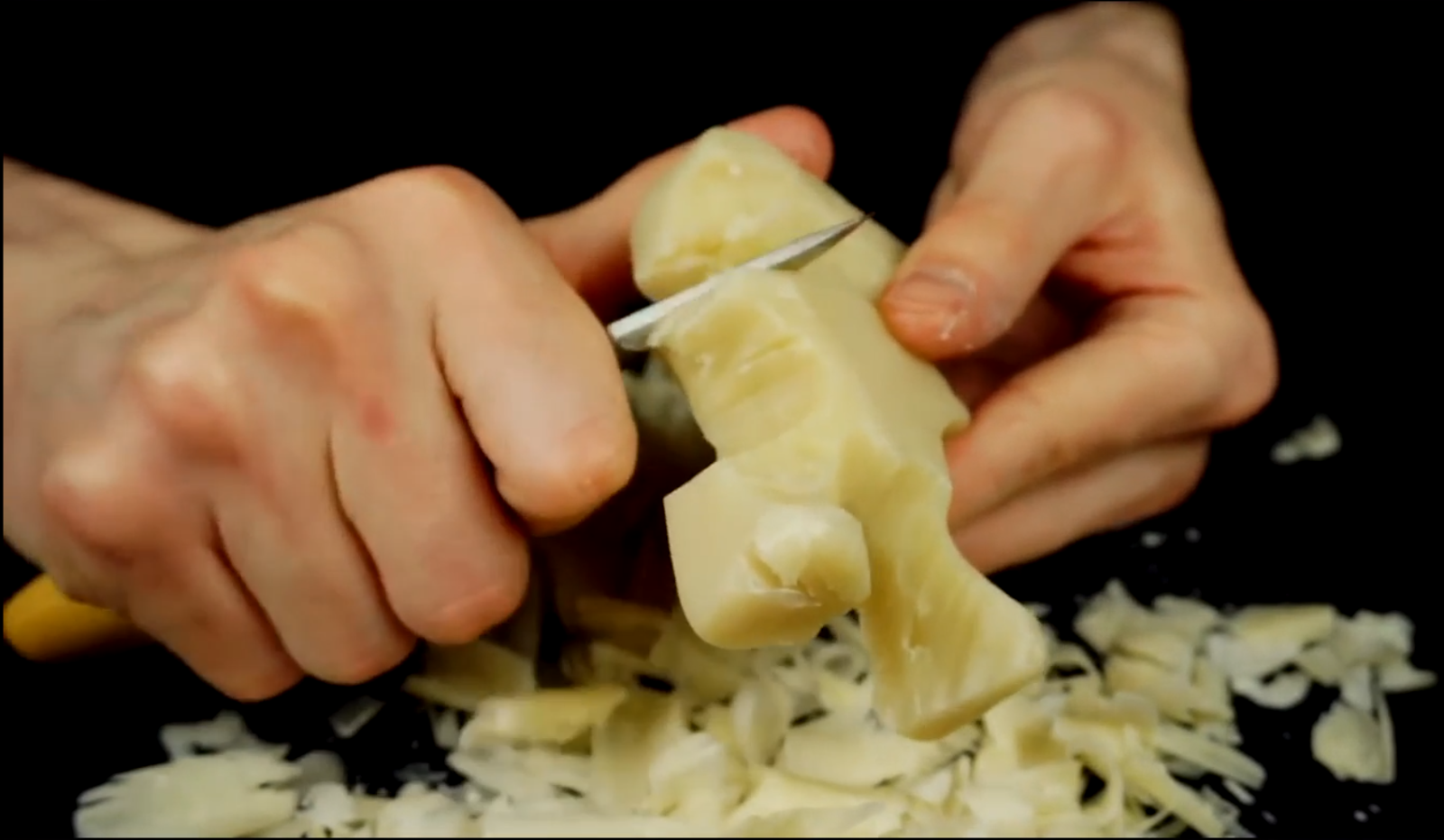 |
| --- | --- | --- |

*Note.* A represents a screenshot from the ASMR video featuring a person eating. This video employs an eye-to-eye format, emphasizing crisp and chewing sounds. B represents a screenshot from the ASMR video showcasing a person playing with water. This video employs a proximity format, highlighting water-dripping sounds. C represents a screenshot from the ASMR video featuring a person carving a soap block. This video employs an attitudinal format, emphasizing scratching sounds.

To ensure that the selected videos are representative of the diverse ASMR videos, we conducted three validations, which makes the selection process more systematic and transparent compared to previous ASMR research (e.g., Eid et al., 2022; B. K. Fredborg et al., 2021). First, we identified videos on YouTube that included ‘ASMR’ in their titles and tags. This indicates that the content creators uploaded these videos intending to induce ASMR.

Secondly, we conducted manual content analysis to ensure that the videos incorporated audiovisual ASMR triggers as previously identified in the literature. The manual content analysis confirmed that these videos indeed contain a significant number of ASMR triggers. According to the ASMR literature (e.g., Barratt & Davis, 2015; Chang et al., 2016; Poerio et al., 2018), a variety of audiovisual ASMR triggers have been recognized. For example, commonly used auditory triggers encompass whispering, crisp sounds (such as the sound of chewing deep-fried food), tapping sounds, scratching sounds, and water-dripping sounds. Visual triggers often involve repetitive motion, slow movement, and various visual formats (e.g., attitudinal, proximity, eye-to-eye) to induce ASMR. Specifically, in the eating video, crisp sounds are present within an eye-to-eye visual format. The water video includes auditory stimuli in the form of water-dripping sounds coupled with the proximal visual format. In the carving video, repeated motions and the sound of a carving knife scratching the soap are incorporated into an attitudinal visual format. The videos were selected based on the most common ASMR triggers identified in prior research (Barratt & Davis, 2015; Poerio et al., 2018), which include whispering, personal attention, crisp sounds (e.g., tapping or eating) and behaviorally, slow movements, and repetitive movements. Although whispering has been consistently reported as the most frequently cited ASMR trigger, it was excluded from this study. Instead, the selected videos featured tapping/scratching sounds (carving video), water/fluid sounds (water video), and eating sounds (eating video). This decision was informed by findings from a Korean sample, which indicated that a majority of Korean participants experienced discomfort or tension in response to whispering (Lee et al., 2019). For this study, we edited these videos into shorter segments (3 minutes each) while retaining the essential ASMR triggers.

Last, we examined how representative (or extraordinary) the selected videos were by conducting a computational analysis of the low-level audiovisual features. This procedure offers insights into the significance of lower-level variables in naturalistic stimuli. Naturalistic stimuli, such as ASMR videos available on YouTube, are multimodal, dynamic stimuli that represent outside-of-the-lab experiences (Sonkusare et al., 2019). Although naturalistic stimuli are becoming increasingly popular as they increase ecological validity (Nastase et al., 2020), they are not without limitations. For instance, the brain response to higher-level audiovisual features (i.e., complex video content) may be confounded with lower-level audiovisual features (e.g., pitch, frequency, hue, saturation, and value; Masson & Isik, 2021). Therefore, we examine the extremity of the selected videos’ low-level audiovisual features (i.e., amplitude and frequency for audio; hue, saturation, and value for visual).

By using a purposefully built Python script, we accessed YouTube using a Selenium Chrome Webdriver that did not have any history or user information. After filtering the search results of ‘ASMR’ by their view counts, we collected 452 ASMR videos. For computational efficiency and constructing a comparable dataset, we randomly extracted 3 minutes from each video. The hue, saturation, value, amplitude, and frequency were calculated for every second of the videos^^[[4]](#footnote-3)^^. As the videos are dynamic, we conducted multidimensional dynamic time warping to compare the similarity between the temporal sequences of video pairs. We calculated the average distance measure for each selected video by comparing it to the 452 video segments. To create a distribution of distance measures, we randomly selected around 100,000 pairs (without replacement) from the pool of 452 video segments.

We examined the likelihood of selecting a pair of ASMR video segments that were more drastically different (i.e., having a higher distance measure) than the average distance measure of the three selected videos. As for the audio features, the average distance measure of the distribution was 3,762 (*SD*= 2,244, minimum = 0, maximum = 18,391, median = 3,302). The average distance measure of the eating video was at the 61st percentile, the water video at the 52nd percentile, and the carving video at the 57th percentile. In other words, more than 39% of randomly paired ASMR videos were more likely to be dissimilar in their low-level audio profiles than the pairs that included the selected videos.

**Figure 2. Distribution of Distance Measures for Low-level Audio Features**


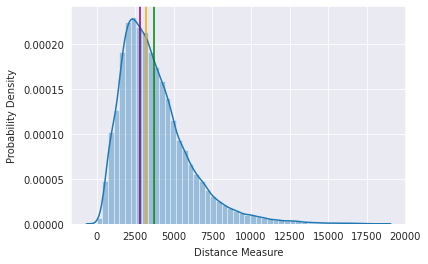


*Note*. The purple line indicates the average distance measure for the eating video, orange for the water video, and green for the carving video.

As for the visual features, the average distance measure of the distribution was 1,157 (*SD* = 541, minimum = 0, maximum = 4,511, median = 1,096). The average distance measure of the eating video was at the 58th percentile, the water video at 77th percentile, and the carving video at 56th percentile. In other words, more than 28% of randomly paired ASMR videos were more likely to be dissimilar in their low-level visual features than the pairs that included the selected videos.

**Figure 3. Distribution of Distance Measures for Low-level Visual Features**


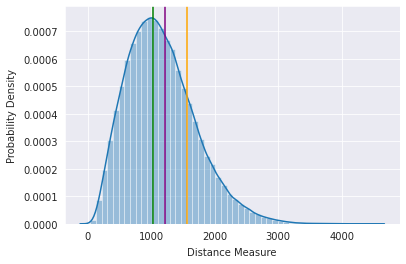


*Note*. The purple line indicates the average distance measure for the eating video, orange for the water video, and green for the carving video.

**References**

Barratt, E. L., & Davis, N. J. (2015). Autonomous Sensory Meridian Response (ASMR): a flow-like mental state. *PeerJ, 3*, article e851. <https://doi.org/10.7717/peerj.851>

Chang, S., Park, J., & Lyou, C. (2016). A study on the existential space in ASMR broadcast. *The Journal of Global Cultural, 24*, 269-287.

Eid, C. M., Hamilton, C., & Greer, J. M. (2022). Untangling the tingle: Investigating the association between the Autonomous Sensory Meridian Response (ASMR), neuroticism, and trait & state anxiety. *PloS ONE*, *17*(2), article e0262668. <https://doi.org/10.1371/journal.pone.0262668>

Fredborg, B., Clark, J., & Smith, S. D. (2017). An examination of personality traits associated with autonomous sensory meridian response (ASMR). *Frontiers in Psychology*, *8*, article 247. <https://doi.org/10.3389/fpsyg.2017.00247>

Lee, J. C., Kim, D., Kim, D. J., Im, S., & Kim, J. Y. (2019). EEG and subjective response to ASMR stimuli. Proceedings of HCIK 2019, 15-18.

Masson, H. L., & Isik, L. (2021). Functional selectivity for social interaction perception in the human superior temporal sulcus during natural viewing. *NeuroImage, 245*(15), article 118741. <https://doi.org/10.1016/j.neuroimage.2021.118741>

Nastase, S. A., Goldstein, A., & Hasson, U. (2020). Keep it real: rethinking the primacy of experimental control in cognitive neuroscience. *NeuroImage*, *222*, article 117254. <https://doi.org/10.1016/j.neuroimage.2020.117254>

Poerio, G. L., Blakey, E., Hostler, T. J., & Veltri, T. (2018). More than a feeling: Autonomous sensory meridian response (ASMR) is characterized by reliable changes in affect and physiology. *PLoS ONE, 13*(6): e0196645. <https://doi.org/10.1371/journal.pone.0196645>

Sonkusare, S., Breakspear, M., & Guo, C. (2019). Naturalistic stimuli in neuroscience: critically acclaimed. *Trends in Cognitive Sciences*, *23*(8), 699-714. <https://doi.org/10.1016/j.tics.2019.05.004>

1. The 3-minute video used in this study is created from the original video. Unfortunately, the original video is no longer available on the content-creator’s channel (<https://www.youtube.com/c/eodyd188>). For those who want to know more about the content of the original video, please see the copy of the original video that was uploaded on a third-party channel: <https://www.youtube.com/watch?v=5IW8tyNBXWs>. The overall content is identical to the original video, but the lighting is darker in this video. [↑](#footnote-ref-0)
2. <https://www.youtube.com/watch?v=THpGyYNnwzs> is the original video uploaded by the content-creator. [↑](#footnote-ref-1)
3. <https://www.youtube.com/watch?v=orD6yIilR0A> is the original video uploaded by the content-creator. [↑](#footnote-ref-2)
4. Crepe (<https://github.com/marl/crepe>), librosa (<https://github.com/librosa/librosa>), and opencv (<https://pypi.org/project/opencv-python/>) are used. [↑](#footnote-ref-3)
